# Survey: acceptability of opportunistic bilateral salpingectomy in Flanders

**DOI:** 10.52054/FVVO.13.3.029

**Published:** 2021-09-24

**Authors:** A.S. Maryns, A Makar, T Hamerlynck, B De Vree, P Tummers, W.A.A. Tjalma

**Affiliations:** Department Gynaecology-Obstetrics, ZNA Middelheim Hospital Antwerp. Lindendreef 1, 2020 Antwerp, Belgium; Women’s Clinic, Ghent University Hospital, Corneel Heymanslaan 10, 9000 Ghent, Belgium; Department Gynaecological Oncology, University Hospital Antwerp; Wilrijkstraat 10, 2650 Edegem, Belgium.

**Keywords:** salpingectomy, carcinoma, ovarian, epithelial, primary prevention, sterilisation

## Abstract

**Background:**

The VVOG (Flemish Society of Obstetrics and Gynaecology) published a consensus statement promoting opportunistic bilateral salpingectomy (OBS).

**Objectives:**

The aim of the study was to obtain insight into the current opinion and general practice of Flemish gynaecologists to counsel and perform OBS.

**Materials and Methods:**

A questionnaire was distributed to Flemish gynaecologists three months after publication of the consensus statement.

**Main outcome measures:**

The drawbacks and incentives to counsel and perform OBS were questioned.

**Results:**

Complete response rate was obtained from 99 gynaecologists (17%) and 37 trainees (19%). The majority of respondents (77%) always counselled for OBS in patients scheduled for hysterectomy without oophorectomy. Eighteen per cent counselled only above a certain age cut off and/or if patient was already menopausal. The most important incentive to counsel in cases of hysterectomy by the abdominal approach and vaginal hysterectomy (VH) was the opportunity to prevent ovarian cancer. The yet-undetermined risk of premature ovarian failure was mentioned as the most important barrier in counselling women for OBS in those undergoing hysterectomy by the abdominal approach. For VH, the respondents saw the risk of complications and increased surgical time as the most important barriers. Sixty-one percent of gynaecologists preferred to perform bilateral salpingectomy as sterilisation method.

**Conclusions:**

Our study suggests that the concept of OBS is already well known in Flanders. There is a positive attitude towards the routine implementation of OBS, although some barriers and doubts about an age cut-off still exist in practice.

## Introduction

The lifetime risk of developing ovarian cancer is 1.4%. About two thirds of cases present with advanced disease stage (FIGO stage III – IV). Despite advances in surgical techniques and anti-tumoral agents, the 5-year survival rate has barely changed over time and is currently at 44%. Approximately 70% of epithelial ovarian cancers are high-grade serous ovarian cancers. The latter can derive from a serous tubal intraepithelial carcinoma (STIC), a precursor lesion in the fimbriae of the fallopian tube. STIC lesions are rare; in Canada salpinges were examined with the SEE FIM protocol (Sectioning and Extensively Examining the Fimbria) and in only 0.38% a STIC lesion was detected (40/10523 specimens) and 80% of STIC lesions were diagnosed with a concurrent gynaecologic cancer (Samimi et al., 2018). The hypothesis is that removal of the fallopian tubes before precursor escape or the development of STIC lesions develop may prevent ovarian cancer. This has led to oncological recommendations to remove the fallopian tubes bilaterally in post-reproductive women already undergoing a surgical procedure, as a primary prevention of ovarian cancer – a procedure called opportunistic bilateral salpingectomy (OBS). The ovary is vascularised through the ovarian artery and through small side branches of the uterine artery. There is concern that salpingectomy could compromise ovarian vascularisation and subsequently could induce premature ovarian failure.

In June 2019, a consensus text of the VVOG – the Flemish Society of Obstetrics and Gynaecology – was published concerning OBS (Tjalma et al., 2019). This consensus statement urges Flemish gynaecologists to counsel all women undergoing hysterectomy for OBS. This consensus also recommends OBS as the preferred method for surgical sterilisation above the age of 40. In cases of early menopause following OBS, hormonal replacement therapy is indicated until the median age of the natural menopause (age 51 years) (Tjalma et al., 2019).

This survey was created to assess whether Flemish gynaecologists and their trainees find OBS to be an acceptable measure in the primary prevention of ovarian cancer. Specific questions addressed the willingness and barriers to performing OBS in combination with hysterectomy as well as utilising OBS as a sterilisation method. Our study aimed to gain insight into the performance of OBS laparoscopically and in combination with a caesarean section.

## Materials and methods

This study was approved by the Ethics Committee of the Ghent University Hospital (reference number: 2019/1066). Study data were collected and managed using REDCap tools (Research Electronic Data Capture). REDCap is a secure, web-based software platform designed to support data capture for research studies. The survey was available online from September to December 2019.

The survey (Appendix, in supplement) consisted of 22 questions. A separate shortened survey of 15 questions was created for trainees in which the questions about the numbers of procedures were removed. All data were collected anonymously. The questionnaires were distributed to the members of the VVOG: 589 certified gynaecologists/ obstetricians and 198 trainees. The hyperlink and QR code to access the survey was disclosed through Gunaikeia (the journal of the VVOG), the VVOG monthly newsletter, the website, email and through the Facebook page of the VVAGO (the Flemish Society of Trainees in Gynaecology and Obstetrics). Additional publicity was undertaken at the national VVOG congress and at compulsory courses for trainees.

All participants were informed about STIC lesions, the risk of STIC and the concept of OBS before they could enter the questionnaire. The survey recorded demographic information, practice pattern questions and the willingness for adopting OBS based on the incentives (possibility to prevent ovarian cancer and the possibility to prevent secondary tubal pathology) and barriers (the need for additional surgical steps and thus fear of increased morbidity, the additional costs, the concern about premature ovarian failure, the increased surgical and counselling time, the lack of evidence and following the habit to leave them behind). The term hysterectomy by abdominal approach was the collective name utilised for total laparoscopic hysterectomy (TLH), laparoscopically assisted vaginal hysterectomy (LAVH) and abdominal hysterectomy (AH).

Analysis was performed with SPSS Statistics version 26. Descriptive tables were separately created for the gynaecologists and trainees, and statistical analysis was performed on the total group of 136 respondents (gynaecologists and trainees).

The weight of the incentives and the barriers for counselling for OBS were questioned with visual analogue scales (VAS) ranging from value 0 to 10 (0 is not relevant and 10 is highly relevant). To detect significant differences between the incentives and barriers for the two approaches (hysterectomy by abdominal approach and vaginal hysterectomy (VH)) a Friedman test was chosen. To compare the approaches, a Wilcoxon signed rank test was performed. To correct for multiple testing, the Bonferroni method was used: the adjusted p-values were cited if they were significant after correction. Boxplots were created to graphically visualise the impact of the arguments.

## Results

Complete response rate was obtained from 99/589 gynaecologists (17%) and from 37/198 trainees (19%). The majority (55%) of gynaecologists were all-round gynaecologists or gynaecologists dealing with benign pathology and worked in non- academic training hospitals. Three-quarters of the trainees were more experienced trainees in their third year (or higher) of training. Characteristics of the two groups can be found in Table I.

**Table I:** Characteristics of the respondents (99 gynaecologists and 37 trainees).

GYNAECOLOGISTS (n (%))					
Work experience in years		Subspecialisation†		Affiliation to centre	
<5	22 (23%)	All-round gynaecologist	55	Academic hospital	20 (20%)
5–15	21 (22%)	Benign gynaecologist	31	Non-academic training hospital	48 (49%)
15–25	25 (26%)	Breast surgeon	16	Non-academic non-training hospital	29 (30%)
>25	26 (27%)	Gynaecological oncologist	11	Other	1 (1.0%)
No longer active*	3 (3%)	Obstetrician	21		
		Urogynaecologist	10		
TRAINEES (n (%))					
Years of training					
First year	5 (14%)				
Second year	5 (14%)				
Third year	8 (22%)				
Fourth year	7 (19%)				
≥ Fifth year	12 (32%)				

*Answers of this subgroup were taken into account; † Multiple answers possible.

### OBS during hysterectomy in general

Ninety-six per cent of the respondents (gynaecologists and trainees) were familiar with the concept of OBS. The majority (77%) always counselled patients, regardless of age or menopausal state, for OBS in cases of planned hysterectomy without oophorectomy. Eighteen per cent counselled for OBS depending on age or menopausal state: 9% only in patients older than 45, 4% only in patients older than 50 and 5% only if patients were deemed menopausal. Only 5% had never counselled patients for OBS in cases of hysterectomy, of whom three participants reported not to be up to date with the theoretical advantage, and four respondents stated not to counsel because OBS was not considered common practice and/or because of limited scientific evidence.

### Incentives and barriers for OBS during hysterectomy by abdominal approach

**Figure 1:**
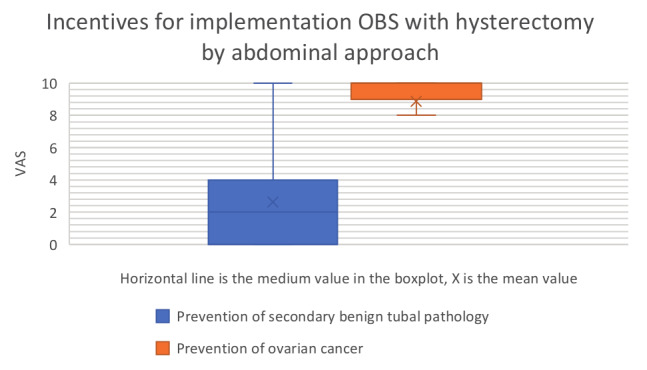
Incentives for the implementation of OBS with hysterectomy by abdominal approach. OBS = opportunistic bilateral salpingectomy.

**Figure 2:**
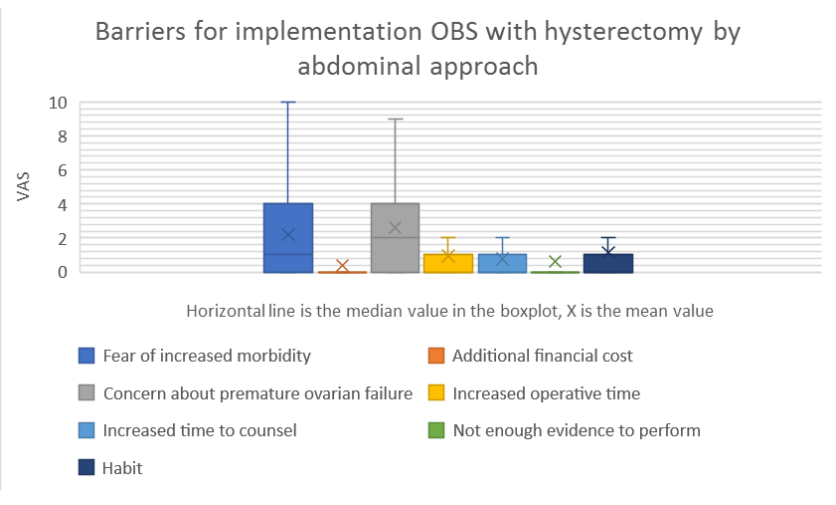
Barriers for the implementation OBS with hysterectomy by abdominal approach. OBS = opportunistic bilateral salpingectomy.

The mean values and standard deviation (SD) of the VAS scores are summarised in Table II and visualised in a box plot (Figures 1 and 2). A possible risk reduction for ovarian cancer (mean 8.87; SD 2.328) was valued as the most important incentive and significantly more decisive than the opportunity to prevent secondary benign tubal pathology (mean 2.63; SD 2.649; p<0.001). The two most important barriers were the concern regarding premature ovarian failure (mean 2.60; SD 2.628) and the fear of increased morbidity (mean 2.19; SD 2.718).

**Table II:** Effect sizes of VAS for incentives and barriers of OBS with hysterectomy by abdominal approach or vaginal hysterectomy.

	By abdominal approach		Vaginal hysterectomy		
	Mean	Standard Deviation	Mean	Standard Deviation	P-value
Incentives					
The prevention of secondary benign tubal pathology	2.63	2.649	2.18	2.553	0.007
The prevention of ovarian cancer	8.87	2.328	7.41	3.459	<0.001
Barriers					
Fear of increased morbidity	2.19	2.718	5.89	3.272	<0.001
Additional financial cost	0.37	1.046	0.44	1.275	0.309
The concern of premature ovarian failure	2.60	2.628	2.10	2.608	0.003
Increased operative time	0.93	1.597	2.45	2.969	<0.001
Increased counselling time	0.75	1.654	0.71	1.549	0.727
Not enough evidence to perform	0.62	1.700	0.74	1.867	0.444
Habit	1.15	2.387	1.330	2.582	0.380

### Incentives and barriers for OBS during VH

**Figure 3:**
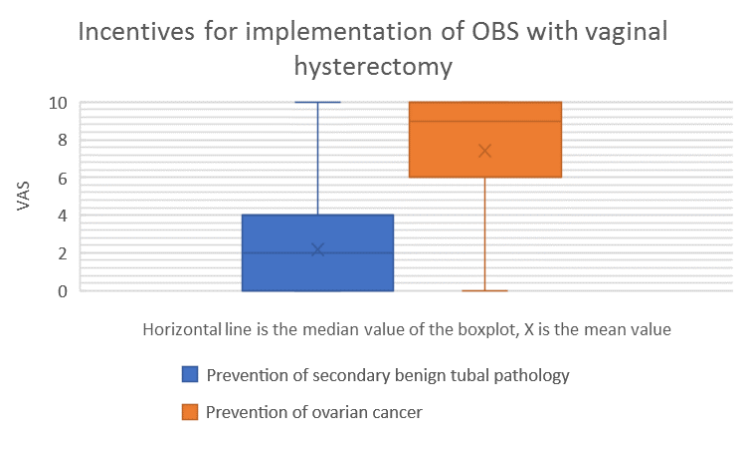
Incentives for the implementation OBS with vaginal hysterectomy. OBS = opportunistic bilateral salpingectomy.

**Figure 4:**
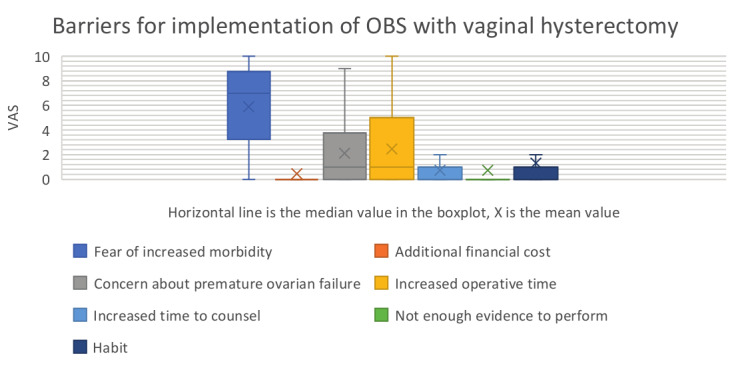
Barriers for the implementation OBS with hysterectomy by abdominal approach. OBS = opportunistic bilateral salpingectomy.

The impact of incentives and barriers for the implementation of OBS with VH are summarised in Table II and a box plot (Figures 3 and 4).

There was a significant difference between the opportunity to prevent ovarian cancer (mean 7.41; SD 3.459) and the opportunity to prevent secondary benign tubal pathology (mean 2.18; SD 2.553; p<0.001).

The most important barrier to perform OBS during VH was the fear of increased morbidity (mean 5.89; SD 3.272), followed by the increased operative time (mean 2.45; SD 2.969) and the concern about premature ovarian failure (mean 2.10; SD 2.608)

### Comparison between hysterectomy by abdominal approach and VH

There was a significant difference for the incentives between the two approaches. Both the opportunity to prevent ovarian cancer (p<0.001) and the opportunity to prevent secondary benign tubal pathology (p=0.007) were valued as more important in the hysterectomy by abdominal approach.

The fear of increased morbidity (p<0.001) and the increased operative time (p<0.001) seemed more relevant with VH, while the concern regarding premature ovarian failure (p= 0.003) was valued as less important vaginally. There were no significant differences between the increased time to counsel (p=0.727), the evidence to perform (p=0.444), the additional financial cost (p=0.309) and the habit not to perform them (p=0.380; Table II).

We also questioned if respondents would change from VH to hysterectomy by abdominal approach if OBS needed to be performed. One-quarter of the respondents would change, but one-quarter also insisted that such a conversion is not needed to perform OBS.

### OBS as sterilisation and during adnexal surgery

Four out of five respondents mentioned OBS as a possible sterilisation technique. In clinical practice, 61% preferred OBS, 23% used clips and 16% performed partial bipolar coagulation. Almost 75% of the respondents agreed to switch to OBS as the general laparoscopic sterilisation technique. Four per cent did not agree and 21% would only agree if age conditions were fulfilled. An age cut-off above 35 years was mentioned (29%), but in most cases the cut-off age quoted was 40 years (52%).

Four out of five respondents (86%) agreed to perform surgical sterilisation in cases where the patient was scheduled for a caesarean section. The preferred sterilisation method during a caesarean procedure was partial tubal resection (55%); less than 29% added OBS. Two-thirds of respondents counselled patients for removal of the contralateral fallopian tube if the patient was planned for unilateral adnexectomy, oophorectomy or salpingectomy. Barriers or incentives were not questioned.

## Discussion

The VVOG published a consensus text for Flemish gynaecologist in favour of OBS (Tjalma et al., 2019). Our survey results, taken less than three months after publication of this text, already reveal a widespread knowledge of the concept of OBS. However, the response rate of the survey was only 17% for gynaecologists and 19% for trainees, which makes it difficult to generalise the results. The response rates of similar online surveys in the Netherlands in 2019 and in Canada in 2013 were 58% and 25%, respectively (Steenbeek et al., 2019; Reade et al. 2013). Compared with surveys about OBS in the UK and Canada, the awareness of STIC lesions was rather high: 96% in Flanders, 90% in Canada and 75% in the UK (Reade et al. 2013; Manchanda et al., 2016).

Two-thirds of the Flemish respondents already discuss OBS with their patients scheduled for hysterectomy. However, in a minority of responders the counselling was influenced by age and menopausal state of the patient. In the Netherlands there is no consensus or guideline about OBS (Steenbeek et al., 2019). A Dutch survey revealed that approximately 70% of respondents counselled patients regarding OBS prior to abdominal hysterectomy and laparoscopic hysterectomy, however only 7% of respondents routinely counselled in conjunction with vaginal hysterectomy and 47% never discussed salpingectomy as a sterilisation method (Steenbeek et al., 2019).

### The opportunity to prevent ovarian cancer

In this survey the main argument to plea for removal of post-reproductive fallopian tubes, was the opportunity to prevent ovarian cancer. The hypothesis for cancer reduction is firstly the removal of the salpinges before precursor lesions of ovarian cancer (STIC lesions) develop in the fimbriae. Secondly salpingectomy could prevent retrograde menstruation which could hypothetically predispose to ovarian epithelial cancer (Tjalma et al., 2019). Falconer et al. (2015) stated, based on a retrospective population-based cohort study, that salpingectomy could reduce the risk of epithelial ovarian cancer. They calculated a hazard ratio (HR) of 0.71 (95% Confidence Interval {CI} 0.56-0.91) for ovarian cancer in case of unilateral salpingectomy and 0.35 (95% CI 0.17-0.73) in case of bilateral salpingectomy. Hysterectomy, unspecified whether with or without salpingectomy, correlated with a HR of 0.79 (95% CI 0.7–0.88) and hysterectomy with bilateral oöphorosalpingectomy with a HR of 0.06 (95% CI 0.03–0.12) (Falconer et al., 2015).

The same Swedish study group used retrospective registry-based data in 2020 on women exposed to pelvic inflammatory disease (PID) to determine the association between PID, with and without secondary salpingectomy, and ovarian cancer risk. There was a more pronounced risk reduction after salpingectomy in women with PID (HR 0.55, 95% CI 0.36-0.83) and the study confirmed their earlier results that salpingectomy, regardless of the indication, reduced ovarian cancer risk, though the effect size appeared lower than the earlier reports (HR 0.72 ,95% CI 0.56-0.63) (Falconer et al., 2020).

Large prospective data on the impact of salpingectomy on ovarian cancer incidence, whether in conjugation with hysterectomy or as a sterilisation method, are still lacking. A meta-analysis of 2017 concluded there were no studies reporting on risk reduction of OBS in conjunction with hysterectomy. While for indicated salpingectomy versus no surgery an adjusted HR of 0.65 (95% CI 0.52-0.81) and adjusted OR of 0.58 (95% CI 0.36-0.95) were calculated (Darelius et al., 2017). The Cochrane review by van Lieshout et al. agreed that the scientific evidence that hysterectomy with OBS results in a reduction of epithelial ovarian cancer is of low to very low quality and long-term results especially in young patients are needed (van Lieshout et al., 2019).

The ongoing Swedish randomised clinical trial, ‘HOPPSA’, comparing hysterectomy with and without OBS will contribute to evidence supporting or contradicting the role of OBS in primary prevention of ovarian cancer after 20 years of follow-up.

### The opportunity to prevent secondary benign tubal pathology

Respondents considered the risk of developing benign tubal pathology (e.g. tubal torsion, formation of hydrosalpinx or salpingitis) if the fallopian tubes are left behind after hysterectomy and the need for a second surgery to be low. However, a Danish study showed that women who have had a hysterectomy without OBS or who have had a laparoscopic tubal ligation had a more than doubled risk of additional tubal surgery (OR 2.13 with 95% CI 1.88–2.42) (Guldberg et al., 2013).

### Concern regarding premature ovarian failure

The Cochrane review of 2019 emphasised that the impact of OBS in combination with hysterectomy on menopausal onset also remains unclear (van Lieshout et al., 2019). Earlier menopause should be avoided at any cost because it is correlated with increased mortality mainly due to cardiovascular diseases (Shuster L et al., 2011).

Trabuco et al. (2016) claimed that hysterectomy by itself could impact ovarian function and shorten the time to menopause. Researchers have tried to gain insight into the impact on ovarian function of adding salpingectomy to hysterectomy, but results are mainly based on surrogate markers of menopause and with short term follow up. Hormonal levels pre- and postoperatively were studied in a small randomised controlled trial: there were no differences in Anti-Mullerian Hormone (AMH) levels 6 months after surgery (van Lieshout et al., 2018). The longest follow up published on surrogate markers was after five years by Venturella et al. They reported no negative effect on ovarian function (based on AMH, follicle-stimulating hormone (FSH), oestradiol levels and ultrasound findings) in the late reproductive years; however, they reported increased menopausal symptoms (Venturella et al., 2017). A retrospective study also calculated an adjusted relative risk (RR) for menopausal symptoms of 1.33 (95% CI 1.04–1.69) one year after hysterectomy with OBS compared to hysterectomy without OBS, and mainly in women aged 44–69 years (RR = 1.53; 95% CI 1.06–2.20) (Collins et al., 2019). However, no adjustments were made for the use of hormonal therapy before surgery. Reassuring results regarding the effect on premature menopause, after adjustment for combined oral anticonception use, were published in the AJOG in 2020. Women who underwent hysterectomy with OBS versus hysterectomy without OBS did not consult earlier for menopausal complaints nor was hormonal replacement therapy initiated sooner (adjusted HR 0.98, 95% CI 0.88-1.09, and adjusted HR 0.82, 95% CI 0.72-0.92 respectively) (Hanley et al., 2020). The ongoing HOPPSA trial will investigate alterations after one year in menopausal symptoms using the validated Menopausal Rating Scale (Idahl et al., 2019).

The age cut-off for salpingectomy as sterilisation method mentioned in the Flemish consensus text and in our survey results, suggests fear of a more negative impact of OBS on ovarian function than ligation or clipping. Hanley et al. (2020) published an adjusted HR of 0.92 (95% CI 0.77-1.10) for the time to consult for menopausal complaints after OBS as sterilisation in comparison with tubal ligation and an adjusted HR 1.00 (95% CI 0.89- 1.12) of the time to start with hormonal replacement therapy. In the Netherlands the Stop Ovarian Cancer Young study is registered on the clinical trial website with registry number NCT04757922. This study will report on the onset of menopause of OBS as sterilisation method. The age of menopause, will be compared between a group of women who had OBS with a control group consisting of women who underwent sterilisation by tubal ligation or who had no sterilisation. If salpingectomy is performed in close proximity to the fallopian tube it seems unlikely that the vascularisation of the ovary would be compromised (Kamran et al., 2013).

In our survey the possibility of premature menopause was valued as more decisive drawback with hysterectomy by abdominal approach than with vaginal hysterectomy. We suppose this might be because in Flanders vaginal hysterectomy is mainly the method of choice for uterine prolapse, most often these are patients already in their late reproductive years, while hysterectomy by abdominal approaches could be more often performed in ‘younger’ patients. But there are no data to support this theory.

### Fear of increased morbidity

Respondents feared increased morbidity mostly if OBS was performed with vaginal hysterectomy, and the fear of increased operative difficulty was also found in other surveys (Cadish et al., 2017; Steenbeek et al., 2019). However, small retrospective and prospective studies have shown that the combination of OBS with vaginal hysterectomy was feasible in 73%–88% of cases (Antosh et al., 2017; Cho et al., 2012; Lamblin et al., 2018). Factors relating to an unsuccessful procedure were postmenopausal state, older age, elevated body mass index and adhesions or fibroids (Antosh et al., 2017; Lamblin et al., 2018). The success rate could be higher due to better exposure if the uterus was first removed and salpingectomy was performed after extraction of the uterus (Cho et al., 2012; Lamblin et al., 2018). The complication rate ranged from 3.8% to 7.95%, being slightly higher in comparison with vaginal hysterectomy without OBS (Cadish et al., 2017; Steenbeek et al., 2019).

One-quarter of the respondents would change from vaginal hysterectomy to a hysterectomy by abdominal approach if OBS has to be performed. A similar trend of conversion was observed in the Dutch survey (Steenbeek et al., 2019). In the United States OBS is performed three times more often with abdominal or laparoscopic hysterectomy than in combination with vaginal hysterectomy (Garcia et al., 2016). V-NOTES (Vaginal Natural Orifice Transluminal Endoscopic Surgery) has gained popularity which makes it possible to perform ‘vaginal’ hysterectomy with salpingectomy more easily without any abdominal scars and OBS as sterilisation could be performed by the same technique (Baekelandt et al., 2016; Chene et al., 2021).

### Sterilisation

The responding Flemish gynaecologists seemed more eager to perform OBS as sterilisation method in comparison with the Dutch colleagues. In the Netherlands, 47% never or rarely counselled patients for salpingectomy as a sterilisation method (Steenbeek et al., 2019). In contrast sixty-one per cent of our respondents agreed to switch to laparoscopic OBS as a general sterilisation technique. Complete bilateral salpingectomy is certainly the most effective method of contraception and secondary tubal pathology is avoided (Deborah and James, 2008; Morse et al., 2006). But as mentioned above, the influence on ovarian function has not been fully assessed.

The Ovarian Cancer Cohort Consortium calculated that bilateral tubal ligation with clips or partial coagulation could lower the risk of epithelial ovarian cancer, mainly a reduction in endometrioid (RR = 0.60; 95% CI 0.41–0.88) and clear cell types (RR = 0.35; 95% CI 0.18–0.69). Hypothesis is that the risk decrease might be due to the prevention of retrograde menstruation. There was only a small reduction in high grade serous carcinoma types following bilateral tubal ligation, which might be hypothetically because the fimbriae are left behind (RR=0.91; 95% CI 0.79 -1.06) (Wentzensen et al., 2016).

To standardise OBS as a laparoscopic sterilisation method, a cut-off age was preferred by 21% of respondents and the age of 40 was most commonly stated. This age limit was also mentioned in the consensus statement (Tjalma et al., 2019). It is impossible to determine if the mentioned cut-off age of our respondents was influenced by the consensus statement or if it was due to the fact that IVF is recommended in women above 40 years instead of an attempt for tubal anastomosis (only possible after clips or tubal ligation) in cases of regret.

Four out of five respondents performed sterilisation in combination with a caesarean section, but less than 29% performed a bilateral salpingectomy. A possible explanation for the ‘low’ number of respondents performing OBS in conjunction with a caesarean section could be the fear of surgical complications, such as bleeding problems (Kamran et al., 2013). However, a systematic review including 320443 patients, revealed no increased rate of surgical complications (Roeckner et al., 2020).

### Strengths and weaknesses

Since there are no data available before publication of the consensus text, it is not possible to estimate the impact on the uptake of OBS in daily practice in Flanders. The response rate of the survey was only 17% from gynaecologists and 19% from trainees, which makes it difficult to generalise the results.

Respondents classified themselves mainly as all-round gynaecologists, which could cause a possible selection bias because non-responder gynaecologists likely have less interest in OBS. Furthermore, most of the trainees were more experienced; this could be explained because first- and second-year trainees do not routinely encounter gynaecological patients while circulating on obstetric wards at the start of their training.

Age and/or menopausal state were questioned for hysterectomy in general but not separately for the two different approaches for hysterectomy (by abdominal approach and VH) and barriers or incentives were not questioned for OBS in combination with adnexal surgery. This was done in an attempt to minimalise the time needed to complete the survey.

### Future perspectives

Since the introduction of consensus statements in other countries, such as Canada, the USA and Sweden, considerably more OBS procedures are performed (Tjalma et al., 2019). Our survey confirmed a willingness and attitude to change current practice. But more high-quality research is needed to confirm or conflict the effect of OBS on ovarian cancer incidence and on the onset of menopause. If future evidence is in favour of OBS, additional training to combine bilateral salpingectomy with caesarean section and with vaginal hysterectomy could be useful or the switch to V-NOTES technique could be made.

Furthermore, adjusted histopathologic analysis of all the resected fallopian tubes could give more insight into the true prevalence of STIC lesions and the effect of our interventions on the risk of ovarian cancer. The timeframe in which STIC lesions develop, and the triggers for spread onto the ovarian surface, are currently not yet known and could vary through the population.

The VVOG consensus text also stated that the implementation of OBS in general practice requires a prospective national registration (Tjalma et al.,2019). While the performance of OBS appears to be widespread already, a registration tool is still lacking.

## Conclusion

Our study suggests that the concept of OBS is already well known in Flanders. Patients are being counselled and OBS is being performed. Furthermore, there is a positive attitude towards the routine implementation of OBS, although some barriers and doubts about an age cut-off still exist in practice. Due to the publication of the VVOG consensus in favour of OBS, the number of procedures may rise over the following years.
